# Editorial: Creating Awareness for Primary Immunodeficiencies in the Southeast and East Asia Regions

**DOI:** 10.3389/fimmu.2022.920819

**Published:** 2022-05-11

**Authors:** Intan Hakimah Ismail, Hirokazu Kanegane, Xiaodong Zhao

**Affiliations:** ^1^ Department of Paediatrics, Faculty of Medicine and Health Sciences, Universiti Putra Malaysia, Serdang, Malaysia; ^2^ Department of Child Health and Development, Tokyo Medical and Dental University (TMDU), Tokyo, Japan; ^3^ Department of Rheumatology and Immunology, Chongqing Medical University, Chongqing, China

**Keywords:** primary immunodeificiency, southeast asia, East Asia, immunology, Nationwide survey, Clinical Immunology

Primary immunodeficiencies (PIDs) are genetic disorders characterized by inborn errors of immunity with various clinical manifestations, including increased susceptibility to infections, autoinflammation, autoimmunity, lymphoproliferation, allergy, and predisposition to malignancy. PID is an emerging disease in several parts of Asia, including East Asia (EA) and Southeast Asia (SEA). Reports from SEA and EA regions demonstrates that PID is more common than usually thought and constitutes an evolving disease that needs to be addressed. However, these disorders receive less attention compared to the other established diseases and lack awareness; therefore, PIDs are often under-reported. The published reports of PIDs in SEA seem to be extremely less than actual case prevalence. This Research Topic is intended to summarize the recent progress in understanding PIDs in SEA and EA regions.

Basic immunology is essential for the understanding of PIDs. DOCK8 deficiency is a combined immunodeficiency characterized by allergic diseases. Jiang et al. revealed that patients with DOCK8 deficiency and the *Dock8* knock-out mouse model had defects in IL-10-producing regulatory B (Breg) cells. In addition, IL-21 restored the function of Bregs in the *Dock8*-deficient mouse.

In patients with Wiskott-Aldrich syndrome (WAS), T-cell receptor (TCR) diversity was severely impaired. Li et al. demonstrated that WASp deficiency selectively affected the TCR diversity of different memory T-cell subsets using *Was* knock-out mouse model.

Infections are the primary cause of morbidity and mortality in patients with PID. Tang et al. demonstrated that metagenomic next-generation sequencing successfully identified microorganism compared with the conventional microbiological test.

A nationwide survey of each country was published on this Research Topic. DNA ligase IV (LIG4) deficiency is an extremely rare PID. Luo et al. described that in patients with LIG4 deficiency, p.R278L variant is observed in most patients and this variant has a founder effect in the Chinese population.

Gastrointestinal (GI) symptoms are often observed in patients with PID. Therefore, Kim et al. performed a nationwide survey of GI symptoms in Korean patients with PIDs. Of 165 patients, 55 (33.1%) showed GI manifestations.

A nationwide survey of PIDs was performed thrice in Japan. Takada summarizes these survey results and focuses on IRAK4 deficiency as an example of creating awareness for its appropriate management.

The Primary Immunodeficiency Database in Japan (PIDJ) is a registry of Japanese patients with PIDs, which was established in 2007. Mitsui-Sekinaka et al. reported that 4,481 patients had been enrolled in PIDJ. In 2017, the Japanese Society for Immunodeficiency and Autoinflammatory Diseases (JSIAD) was launched. Furthermore, the PIDJ was upgraded to “PIDJ ver.2” in 2019, supported by JSIAD to promote epidemiological studies, genetic analysis, and pathogenetic evaluation for PIDs and autoinflammatory diseases.

Serum immunoglobulin (Ig) measurements are not widely accessible in developing countries. Suratannon et al. reported a simple prediction model using serum globulin to predict the likelihood of low IgG levels in children.

PIDs may have phenotypic differences in the SEA, EA, and other regions. For example, Kadowaki et al. described a characteristic of A20 haploinsufficiency (HA) in Asia. HA20 is an early-onset autoinflammatory disease resembling Behçet’s disease (BD). Patients with HA20 in the EA developed recurrent fever more frequently than those in other regions but were less likely to develop BD symptoms.

Some patients with early-onset inflammatory bowel disease (IBD) are associated with PIDs. Sasahara et al. review PID-associated early-onset IBD in SEA and EA. The prevalence of PIDs associated with IBD was higher than in western countries. A Japanese cohort revealed that patients with XIAP and IL10RA deficiencies were recurrently observed; however, patients with IL10R deficiency were preferentially reported in China. Therefore, a comprehensive molecular diagnosis should be applied to screen for PID-associated IBD in SEA and EA to improve the prognosis.

Chronic granulomatous disease (CGD) is a PID characterized by recurrent bacterial and fungal infections and it is inherited as an X-liked (XL) or autosomal recessive (AR) mode. Chiu et al. revealed phenotypic differences between XL- and AR-CGD in Asia and Africa. XL-CGD patients had a younger age of onset, referral, and diagnosis than AR-CGD patients. In addition, patients with XL-CGD had more recurrent and severe infections than patients with AR-CGD.

A couple of comprehensive reviews of PIDs are included in this Research Topic. Miyazawa and Wada discussed the most recent research on reversion mosaicism in PIDs. Several mechanisms can mediate the somatic reversion of inherited variants. Furthermore, revertant cells with wild-type function may be associated with milder phenotypes in PID patients with reversion mosaicism.

IKAROS and CTLA4 deficiencies are PIDs showing similar clinical phenotypes, including hypogammaglobulinemia and autoimmune disease (AD). Hoshino et al. performed a systematic literature review of these diseases. IKAROS deficiency revealed that AD and hypogammaglobulinemia develop in that order, and AD resolves before the onset of hypogammaglobulinemia. Conversly, these observations were not found in CTLA4 deficiency.

PIDs frequently affect the endocrine system. Takasawa et al. comprehensively reviewed the understanding of endocrine disorders clinical features and pathophysiology in PIDs.

This Research Topics included interesting case reports. For example, Ripen et al. reported on a Malaysian girl affected with both Williams-Beuren syndrome and CGD (*NCF1*). Whole-exome sequencing revealed a hemizygous deletion of 1.53Mb on chromosome 7q11.23.

Inaba et al. presented a report of a Japanese boy with NEMO deficiency caused by a novel hypomorphic variant of *IKBKG*. He also stated that his deceased maternal uncles had the same *IKBKG* variants using preserved umbilical cord blood cells.

Severe combined immunodeficiency (SCID) is a PID primarily due to impaired lymphocyte differentiation. Hematopoietic cell transplantation (HCT) is a curable treatment, but the outcomes of HST for SCID are poor when patients have active infections. Tanita et al. reported that five Japanese patients with SCID were infected with rotavirus infection derived from the oral rotavirus vaccine.

Li et al. described six Chinese patients with type I interferonopathy who were treated with Janus kinase (JAK) inhibitors. The JAK inhibitors (baricitinib and tofacitinib) are promising drugs for patients with type I interferonopathy including STING-associated vasculopathy with onset in infancy, Aicardi-Goutières syndrome, and spondyloenchondrodysplasia with immune dysregulation.

## Author Contributions

HK wrote the manuscript. II and XZ provided critical discussion. IHI and XZ edited the manuscript. All authors contributed to the article and approved the submitted version.

## Conflict of Interest

The authors declare that the research was conducted in the absence of any commercial or financial relationships that could be construed as a potential conflict of interest.

## Publisher’s Note

All claims expressed in this article are solely those of the authors and do not necessarily represent those of their affiliated organizations, or those of the publisher, the editors and the reviewers. Any product that may be evaluated in this article, or claim that may be made by its manufacturer, is not guaranteed or endorsed by the publisher.

